# Unusual Presentation of Gianotti-Crosti Syndrome due to Epstein-Barr Virus Infection

**DOI:** 10.1155/2016/1017524

**Published:** 2016-12-05

**Authors:** Hind Saif Al Dhaheri, Amani Al Kaabi, Yasmin Kara Hamo, Aysha Al Kaabi, Salwa Al Kaabi, Hossam Al Tatari

**Affiliations:** Department of Pediatrics, Tawam Hospital, Al Ain, UAE

## Abstract

Gianotti-Crosti syndrome (GCS) is viral exanthema of childhood. It typically presents with a symmetric erythematous papular and papulovesicular eruption. It has been classically associated with hepatitis B virus, as well as rarely with Epstein-Barr virus (EBV). We report a case of GCS related to EBV infection without the classical systemic symptoms in a five-year-old male patient.

## 1. Introduction

Gianotti-Crosti syndrome (GCS) is a viral exanthem of childhood that appears most commonly in children between the ages of 1–6 years but also reported in other age groups between 3 months to 15 years [1].

The most common causative agent is hepatitis B virus (HBV). Other agents include hepatitis C and hepatitis A, influenza, parainfluenza, adenovirus, cytomegalovirus (CMV), Epstein-Barr virus (EBV), and respiratory syncytial virus (RSV) [2]. GCS was also associated with vaccine administration [3] such as oral polio vaccine, pentavalent vaccine, Diphtheria, Pertussis, and Tetanus (DPT), hepatitis B and haemophilus influenza type b, and hepatitis A vaccine. The mean time of developing the rash ranges from 2 days to 21 days [4].

This viral exanthem typically consists of symmetrical erythematous papular or vesiculopapular eruptions with an acral spreading. It frequently starts from the buttocks and spreads to other areas of the body [5, 6]. GCS is a clinical diagnosis and the treatment is supportive since it is a self-limited disease [1, 5].

## 2. Case Report

A previously healthy 5-year-old male was admitted to the paediatric medical ward due to 2-day history of blood tinged diarrhoea and 2-week history of abdominal pain with itchy rash. No fever, joint pain, cough, nasal congestion, or urinary symptoms were reported at that time. He was fully vaccinated and his family medical history was completely negative.

On physical examination, he had normal growth parameters with normal vitals. Systems examination was unremarkable apart from symmetrical itchy erythematous maculopapular rash over the extensor surfaces of upper and lower limbs. The rash was mainly concentrated over elbows and ankles. No lymphadenopathies, hepatosplenomegaly, or joints involvement was appreciated at that time.

The rash morphology and distribution were suggestive of GCS and therefore he was investigated accordingly. Investigations revealed the following results: complete blood count showed normal WBC with monocytosis (1.2 × 10^9^/L); renal and liver function tests were within normal range for the age; and urinalysis was normal. Hepatitis Bs Ag was negative. Despite the lack of typical mononucleosis syndrome, EBV infection was suspected due to the significant monocytosis in the CBC. Therefore, EBV PCR was checked and turned to be positive. He was provided with symptomatic care and was discharged after two days in good condition. The patient was followed up regularly in the outpatient clinic and the rash was found to fade completely by the end of the fifth week of illness.

## 3. Discussion

Gianotti-Crosti syndrome (GCS) is a viral exanthem of childhood that was first reported by Ferdinando Gianotti and Agostino Crosti in 1957 as a monomorphous erythematous rash of infants and children [1, 5].

Incidence of GCS is not well defined although many believe that it is an underdiagnosed condition. It appears most commonly in children between the ages of 1–6 years. There are also few case reports in children as young as 3 months and as old as 15 years of age. In paediatric population there is no statistical differences in gender or race [1]. GCS has higher incidence during spring and summer [2] and in patients with personal or family history of atopy [1].

The pathogenesis of GCS is not clear; however studies had reported two main hypotheses; the first was IgE mediated response supported by the fact that GCS is seen more in patients with atopy. The second hypothesis was viral induced delayed type hypersensitivity reaction due to high CD4^+^ T cell counts in the dermal infiltrate of affected patients [1, 5].

Case reports have clearly indicated the association between infectious triggers and development of GCS. Most common causative agent is HBV; other agents include hepatitis C and hepatitis A (HAV), EBV, influenza, parainfluenza, adenovirus, CMV, and RSV [2]. Though rare, bacterial causes have been reported as well, including* Bartonella henselae*, b-hemolytic streptococci,* Borrelia burgdorferi*, and* Mycoplasma pneumonia* [1, 5]. GCS was also reported after vaccine administration such as influenza virus vaccine, HBV and HAV vaccine, oral polio vaccine, and measles-mumps-rubella vaccines; however no causal correlation has been recognized [3, 5, 7]. In our case GCS was confirmed to be caused by EBV.

GCS main clinical feature is a viral exanthem, which might be preceded by upper respiratory infection, diarrhoea, or pharyngitis. The rash of GCS is characterized by symmetrical erythematous papular or vesiculopapular eruption with an acral spreading, frequently starting from the buttock and spreading acrally [4, 5]. Other areas of the body (trunk, elbows, knees, palms, and soles) are usually not affected; however its involvement does not exclude the diagnosis. The size of the rash ranges between 1 and 10 millimetres. Most children will have spontaneous resolution of the rash in 10 days to 6 months. However there are reports of the rash lasting for as short as 5 days and as long as 12 months. Mild to severe pruritus might be present and may last up to several weeks. In our case the rash was itchy and distributed over the extensor surfaces of upper and lower limbs while being concentrated around the knees and elbows, which is typical for GCS.

Systemic manifestation associated with GCS includes low-grade fever, malaise, diarrhoea, and lymphadenopathies (25–35% of patients). Hepatitis is rare and is mainly seen with HBV, EBV, and CMV infection in the form of anicteric hepatitis. Splenomegaly was rarely reported [1, 5]. Our patient was unique in a way since he did not have any of the above manifestations despite having positive blood EBV PCR. His blood tinged diarrhoea could be explained by the chronic constipation that he had and recent use of fleet enema.

GCS is a self-limited benign disease that could be puzzling to paediatricians and disturbing to many parents. Reaching the diagnosis and educating the parents about it will surely help in resolving parents' anxiety. For pruritus, treatment depends on the severity and ranges from topical emollient to oral antihistamine [1, 5].

## 4. Conclusion

By reporting this unique case we encourage paediatricians to consider GCS in their differential diagnosis of viral exanthems and to consider EBV as an underlying cause even in the absence of the typical features of EBV infection.

## Abbreviations

GCS: Gianotti-Crosti syndrome
EBV: Epstein-Barr virus
HBV: Hepatitis B virus
HAV: Hepatitis A virus
CMV: Cytomegalovirus
RSV: Respiratory syncytial virus
HSP: Henoch-Schönlein purpura.
